# Exploiting Human NK Cells in Tumor Therapy

**DOI:** 10.3389/fimmu.2019.03013

**Published:** 2020-01-17

**Authors:** Paola Vacca, Gabriella Pietra, Nicola Tumino, Enrico Munari, Maria Cristina Mingari, Lorenzo Moretta

**Affiliations:** ^1^Immunology Research Area, IRCCS Bambino Gesù Pediatric Hospital, Rome, Italy; ^2^UOC Immunology, IRCCS Ospedale Policlinico San Martino, Genoa, Italy; ^3^Department of Experimental Medicine (DIMES), Università di Genova, Genoa, Italy; ^4^Department of Pathology, IRCCS Sacro Cuore Don Calabria, Negrar, Italy; ^5^Department of Experimental Medicine (DIMES), Center of Excellence for Biomedical Research, Università di Genova, Genoa, Italy

**Keywords:** NK cells, inhibitory checkpoints, innate immunity, immunotherapy, anti-tumor therapy

## Abstract

NK cells play an important role in the innate defenses against tumor growth and metastases. Human NK cell activation and function are regulated by an array of HLA class I-specific inhibitory receptors and activating receptors recognizing ligands expressed *de novo* on tumor or virus-infected cells. NK cells have been exploited in immunotherapy of cancer, including: (1) the *in vivo* infusion of IL-2 or IL-15, cytokines inducing activation and proliferation of NK cells that are frequently impaired in cancer patients. Nonetheless, the significant toxicity experienced, primarily with IL-2, limited their use except for combination therapies, e.g., IL-15 with checkpoint inhibitors; (2) the adoptive immunotherapy with cytokine-induced NK cells had effect on some melanoma metastases (lung), while other localizations were not affected; (3) a remarkable evolution of adoptive cell therapy is represented by NK cells engineered with CAR-targeting tumor antigens (CAR-NK). CAR-NK cells complement CAR-T cells as they do not cause GvHD and may be obtained from unrelated donors. Accordingly, CAR-NK cells may represent an “off-the-shelf” tool, readily available for effective tumor therapy; (4) the efficacy of adoptive cell therapy in cancer is also witnessed by the αβT cell- and B cell-depleted haploidentical HSC transplantation in which the infusion of donor NK cells and γδT cells, together with HSC, sharply reduces leukemia relapses and infections; (5) a true revolution in tumor therapy is the use of mAbs targeting checkpoint inhibitors including PD-1, CTLA-4, the HLA class I-specific KIR, and NKG2A. Since PD-1 is expressed not only by tumor-associated T cells but also by NK cells, its blocking might unleash NK cells playing a crucial effector role against HLA class I-deficient tumors that are undetectable by T cells.

## Introduction

Natural killer (NK) cells play a central role in innate defenses against viruses and tumors. They belong to a family of innate lymphoid cells (ILC) that do not express receptors encoded by rearranged genes. NK cell function is regulated by an array of inhibitory and activating receptors. Inhibitory receptors that play a major role in the control of NK cell function are those specific for HLA-class (Cl)-I molecules. Killer Ig-like receptors (KIRs) recognize allotypic determinants shared by different HLA-Cl-I alleles, while CD94/NKG2A is specific for the non-classical HLA-E (1). The fact that NK cell inactivation is required to spare healthy cells implied the existence of activating receptors recognizing ligands on target cells. The prototypes and most important ones in tumor cell detection and killing are NKp46, NKp44, and NKp30, collectively called “natural cytotoxicity receptors” (NCRs) (2). While in an autologous setting all NK cells express one or more inhibitory receptors for self HLA-Cl-I, in an allogeneic setting, it may occur that KIRs expressed by a subset of NK cells do not recognize HLA-Cl-I alleles on allogeneic cells, and the lack of inhibition may result in killing of target cells (3). NK cells with these characteristics were named “alloreactive” NK cells. Although NK cells display a potent anti-tumor activity and are thought to participate to the control of tumor growth and metastatic spread, the tumor microenvironment may sharply inhibit their effector functions (4, 5). This inhibitory effect is due to the tumor cells themselves as well as to other cells present in the tumor microenvironment and frequently involves both the downregulation of activating surface receptors and the *de novo* expression of inhibitory checkpoints (primarily PD-1) (6, 7). In this contribution, we will briefly discuss different therapeutic strategies (Table 1), which allow to successfully exploit NK cell-mediated anti-tumor activity as well as novel promising approaches that may offer important new tools in cancer treatment.

**Table 1:** Human NK cell-based immunotherapeutic approaches in tumors.

**1. Adoptive NK cell therapies** - Infusion of IL-2- or IL-15-activated NK cells or lymphokine-activated lymphocytes (LAK and CIK) (8–11); - Infusion of allogeneic “off-the-shelf” CAR-NK cells directed to tumor antigens (12).
**2. NK cells in haplo-HSCT to cure high-risk leukemia** - Transplant of “pure” donor CD34^+^ cells. NKG2A^+^ NK cells are detectable after 2 weeks, while KIR^+^, cytolytic NK cells only after 6–8 weeks. Central role of NK cells in GvL, especially of “alloreactive” NK cells (13, 14); - Transplant of *αβ*T- and B cell-depleted mononuclear cells. Donor NK cells and *γδ*T cells, being present in the graft, are immediately available for the control of infections and leukemia relapses. Better clinical outcome, particularly in AML (15–19).
**3.mAbs blocking inhibitory checkpoints in NK cells** - The disruption of PD1/PD-L1 interactions unleashes both PD1^+^ T and NK cells. Major effect of NK cells in case of HLA-Cl-I^−^ tumors (20–24); - Blocking of NKG2A expressed by both NK and tumor-infiltrating T cells results in killing of HLA-E^+^ tumors (i.e., most tumors) (25, 26); - Combined blocking of NKG2A and PD1 in case of PD-L1^+^ tumors (25, 26); - Combined use of NKG2A-blocking mAb and mAb specific for tumor antigens (e.g., EGFR): “unlocked” NK cells mediate ADCC (25, 26).


## Boosting *in vivo* NK Cells With Immune Stimulatory Cytokines

In cancer patients, NK cells frequently display an impaired function (6, 27). Thus, primary strategies in immunotherapy are aimed to boost *in vivo* NK cell-mediated antitumor activity. One approach is based on the *in vivo* administration of cytokines, such as IL-2 and IL-15, that determine NK cell activation, differentiation, and expansion (8, 28–32). IL-2 administration was approved in the 1990s for the treatment of metastatic RCC and melanoma patients (33–35). Two major obstacles in IL-2-based therapy are the dose-associated toxicity (primarily vascular leakage) and the induction of T regulatory (Treg) cell activation and expansion, thus resulting in inhibition of NK cell function (9, 10). Recently, IL-2 variants, with lower affinity for IL-2Rα subunit (highly expressed by Treg cells), have been designed (11, 36, 37). In addition, PEGylated IL-2 (also known as NKTR-214) that binds CD122 (IL-2Rβ), expressed by both T and NK cells, is able to boost preferentially these cells and their anti-tumor responses. This therapeutic treatment is currently under investigation in clinical trials for solid tumors (13). The use of IL-15 may represent a better therapeutic option as it can selectively sustain NK cells without inducing Treg expansion. However, the clinical use of IL-15 is limited because of its short *in vivo* half-life (38). Notably, IL-15 induces a rapid expansion of memory CD8^+^ T cells, thus favoring anti-tumor activity. The effect of IL-15 administration combined with checkpoint inhibitors (anti-CTLA-4 and/or anti-PD-1 mAbs) is currently under investigation in patients with cancers refractory to other therapies. To improve the anti-tumor effect of NK cells, ALT-803, an IL-15 superagonist complex, is also being assessed in phase I studies either alone (14) or in combination with checkpoint inhibitors (39). An emerging approach is based on bi- or tri-specific killer cell engagers (BiKEs and TriKEs) binding CD16 or NKG2D on NK cells and tumor antigens, thus favoring the interaction between NK cells and tumor cells. Notably, “TriKEs” also contain a modified IL-15 linker to improve NK cell survival and proliferation (15, 40, 41). An additional prospect is the use of IL-12, a molecule that enhances cytokine production and cytotoxicity by NK cells (16).

## Adoptive Immunotherapy With Cytokine-Induced NK Cells

Clinical trials have been attempted since 1980s in which NK cell-containing cell suspensions isolated from patients with metastatic melanomas were expanded *in vitro* in the presence of IL-2 and infused back into the patients. While a relevant effect was detected in some cases, primarily in metastatic lesions such as lung metastases, other tumor localizations were not affected. These studies were important because they provided the first evidence that such “lymphokine-activated killers” (LAK) could exert their anti-tumor effect also *in vivo*. Relevant toxicity was mostly related to the concomitant administration of high dosages of IL-2 (17, 18). Evolutions of such pioneering studies, based on adoptive cell therapy, were the use of IL-15 and, recently, the use of NK cells engineered with chimeric antigen receptor (CAR, see below). Although NK cells to be used in adoptive tumor therapy are usually derived from peripheral blood (PB), other sources have also been proposed. For example, the pleural fluid of primary or metastatic tumors contains high numbers of functional NK cells (19), which acquire strong cytotoxicity upon short culture intervals with IL-15 or IL-2 *in vitro*. Since large volumes of such fluids are routinely discarded, NK cells could be recovered and reinfused systemically or in the pleural cavity after *in vitro* culture with IL-15 (8). Such loco-regional treatment might contribute to the control of pleural localizations of the tumor. In general, the infusion of potent effector cells with anti-tumor activity is an important approach in tumor immunotherapy because it may greatly amplify the effect of endogenous cells. The relevance of infusing mature effector cells in cancer patients is also underscored in the αβT- and B-cell-depleted haploidentical hematopoietic stem cell transplantation (HSCT), in which leukemia relapses and infections are sharply reduced as compared to the HSCT setting with “purified” CD34^+^ cells, thanks to the co-infusion of mature γδT cells and NK cells (see below).

## Role of NK Cells in the Therapy of High-Risk Leukemia in Haploidentical HSCT

HSCT represents the life-saving therapy for acute leukemia poorly responsive to chemotherapy, relapsing, or with adverse cytogenetic characteristics. Unfortunately, it is possible to find a HLA compatible donor only for ~60% of patients (42, 43). Thus, T-depleted haplo-identical HSCT has been developed in an attempt to rescue those patients for whom no alternative therapeutic option is available. Haplo-HSCT is based on the infusion of “megadoses” of purified CD34^+^ cells extensively depleted of T cells in order to avoid life-threatening GvHD. In this transplantation setting, donor NK cells may express KIR that do not recognize any of the HLA-Cl-I alleles of the patient (44, 45). Notably, NK cells are the first donor lymphoid cells detectable in patients' PB after transplantation. In pediatric patients, this occurs after ~2 weeks. However, such NK cells are represented by relatively immature CD56^bright^ cells, expressing NKG2A but not KIR (KIR expression is required for NK cell alloreactivity). Appearance of mature KIR^+^ NK cells requires an additional 4–6 weeks. In this T-depleted HSCT setting, NK cells play a major role in graft-vs.-leukemia (GvL) (46). The anti-leukemia effect has been related to NK cell alloreactivity in different studies, pioneered by Ruggeri et al., in adult AML (20, 21, 45). Indeed a clear correlation was found between the frequency of alloreactive NK cells and the clinical outcome (22, 44). Of note, a subset of NK cells derived from CMV-seropositive donors could undergo expansion in transplanted patients upon CMV reactivation after HSCT. These NK cells expressed NKG2C, CD57, and displayed epigenetic modifications identical to those present in memory T cells. These characteristics confer to NK cells a strong cytolytic activity, including a “memory-like” behavior in response to NKG2C triggering, and are associated to a better clinical outcome (23–25, 47, 48). In pediatric patients receiving “megadoses” of purified CD34^+^, the survival probability at 5 years was very good for patients with high-risk ALL, reaching over 70% in the presence of NK alloreactivity and ~40% in its absence, the overall survival being ~60%. In patients with AML, survival reached ~40% in case of NK alloreactivity, but only ~20% in its absence, the overall survival being ~30%. Notably, all deaths occurred early, during the first few weeks/months after transplant, primarily due to leukemia relapses or infections (20, 49). In an attempt to fill the temporal gap between transplant and the generation of mature KIR^+^ alloreactive NK cells, a novel graft manipulation has been developed. This is based on the selective depletion of TCR αβ T cells (responsible of GvHD) and B cells (to prevent B cell malignancies in immunocompromised individuals). With this graft manipulation, the infused mononuclear cells also contain, in addition to HSC (including not only CD34^+^ but also CD34^−^ precursors), effector cells such as mature (CD56^dim^) NK cells and TCRγδ T cells, both capable of anti-leukemia activity (12, 26, 50). In addition, the graft contained different myeloid cell types, including monocytes and low-density monocytic or polymorphonuclear (PMN) myeloid cells (51, 52). The immediate availability of cells capable of killing leukemia blasts and controlling virus reactivation or infections had a major positive impact. Indeed the overall survival probability was ~70% not only for ALL but also for AML patients. An unexpected finding was that NK cell-mediated alloreactivity did not appear to play a significant role (49). While it is conceivable that the NK cell function may be offset by a predominant GvL effect of γδT cells (greatly expanded *in vivo* thanks to the use of zoledronic acid) (53), we could not exclude that also other mechanisms may impair NK alloreactivity. Indeed we recently found that myeloid-derived suppressor cells (MDSC), particularly abundant in the graft, exert a potent inhibitory effect on NK cell function (54). These data suggest a possible effect also *in vivo* and offer a clue for applying an additional step in the graft manipulation to further remove MDSC. The rescue of NK cell function may contribute to increase the clinical outcome, particularly by preventing leukemia relapses, still representing ~25% of total deaths.

Taken together, these data support the notion that NK and other cells of the innate immunity may play a relevant role in the therapy of high-risk leukemia. Notably, HSC from different sources give rise to other innate lymphoid cells (ILC), particularly ILC3. ILC3 cells contribute to tissue repair and regeneration of lymphoid tissues and are likely to play a major role in the integrity of such tissues severely compromised by the chemo/radiotherapy given to patients prior to HSCT (55).

## Blocking of Inhibitory Checkpoints/Receptors to Unleash NK Cells

NK cells express inhibitory receptors such as the HLA-Cl-I-specific KIRs and CD94/NKG2A that may function as true inhibitory checkpoints (56). The lack of interactions with their cognate HLA class I ligands on target cells leads to cytolytic activity and cytokine production. This may occur in an autologous environment in the case of tumors or viral infections, as well as in an allogeneic setting such as the haplo-HSCT (see above).

While KIRs and NKG2A are constitutively expressed by mature NK cells, the expression of other inhibitory checkpoints involved in the homeostasis of immune responses, including PD-1, TIGIT, TIM-3, and CD96, is inducible (57). Such *de novo*-expressed checkpoint regulators have been shown to inhibit the NK cell function upon interaction with their ligands on tumor cells (19, 58). We will focus on PD-1 since it is a major checkpoint receptor involved in the control of immune responses, and the therapeutic use of blocking antibodies disrupting the PD-1/PD-L1 axis represents a major breakthrough in the cure of highly aggressive tumors.

While PD-1 expression has been first reported in T lymphocytes, recent studies revealed that, in pathological conditions, such as CMV infections and tumors, it may be expressed also by NK cells. The expression of PD-1 by NK cells is controversial; indeed PB-NK cells derived from both healthy donors (HD) and neoplastic patients were originally reported to express low levels, if any, of PD-1. On the other hand, PD-1^bright^ NK cells were found in the PB and, more abundantly, in ascitic fluid of ovarian carcinoma patients (58), as well as in pleural effusions of patients with primary and metastatic tumors (19) and in Hodgkin lymphoma. Notably, both PD-1 mRNA and PD-1 protein are present in the cytoplasm of NK cells isolated from HD (59), although the molecular mechanisms leading to its surface expression are still poorly defined.

Under physiological conditions, PD-1 acts as a brake in the regulation of immune responses, playing a relevant role in the induction and maintenance of T cell tolerance. However, in cancer patients, it may impair T cell- and NK cell-mediated responses against tumor cells. In these cases, immunotherapy with mAbs disrupting the PD-1/PD-L1 interaction has shown great effectiveness, particularly in melanoma and lung carcinomas with responses to therapy reaching 20–40% in different clinical trials. Importantly, therapeutic blockade of inhibitory checkpoints in NK cells may be effective also in HLA-Cl-I^neg^ tumors, a condition that frequently occurs in metastatic carcinomas (as a result of tumor escape from cytolytic T cell-mediated control) (60, 61). Nevertheless, the majority of patients do not benefit from the anti-PD-1/PD-L1 treatment. Thus, prediction of clinical responses to PD-1/PD-L1 blockade represents a major issue also in view of important side effects and of the high treatment cost. In this context, an important approach is the evaluation of PD-L1 expression on tumor cells. However, its predictive value is still unsatisfactory due to several technical limitations, such as the use of different mAbs, different diagnostic materials (biopsies vs. surgical specimen, cytology), and different operators (62–64). For this reason, current researches are aimed to identify additional checkpoints to be targeted, either alone or in combination. In this context, the actual potential of blocking TIGIT, TIM-3, CD96, or LAG-3 is currently under investigation. Importantly, a recent study by Vivier's group has highlighted the use of anti-CD94/NKG2A blocking mAb in tumor therapy (65). NKG2A^+^ cells represent >50% of PB-NK cells and may express either the CD56^bright^ or the CD56^dim^ phenotype. While CD56^bright^ NKG2A^+^ NK cells are primarily cytokine producers, CD56^dim^ NKG2A^+^ cells also display potent cytolytic activity and DC editing capability (66). NKG2A is also expressed by T lymphocytes, either upon prolonged stimulation via TCR (67) or upon exposure to TGF- β (68), an immunosuppressive cytokine often present in the tumor microenvironment. This *de novo* NKG2A expression may lead to the impairment of T cell function, including anti-tumor activity (67). Accordingly, blocking of NKG2A can unleash not only NK cells but also tumor-infiltrating T cells with potential anti-tumor activity. In addition, HLA-E, the NKG2A ligand, is expressed in many highly aggressive tumors (e.g., lung, head and neck, colon, pancreas, and liver), and most cells in the tumor are HLA-E^+^. Accordingly, blocking of NKG2A may result in potent anti-tumor effect in different cancers. In tumors expressing both HLA-E and PD-L1, the combined blocking of NKG2A and PD-1/PD-L1 axis can enhance NK cell cytotoxicity and rescue T cell function. Notably, in a murine model, this combined treatment also resulted in T cell proliferation and T cell memory induction. Finally, in HLA-E^+^ tumors, expressing tumor-associated antigens, NKG2A blockade could increase the therapeutic efficacy of other mAbs (for example, anti-EGFR mAb), favoring the NK cell triggering via the CD16-mediated antibody-dependent cytotoxicity (ADCC) (65, 69). These different scenarios involving NKG2A blockade are promising because they may occur in many tumors and involve important synergies with other checkpoint inhibitors or therapeutic antibodies directed to tumor antigens. In addition, these studies emphasize the importance of harnessing NK cell-mediated anti-tumor activity while, so far, the immunotherapeutic strategies have been mostly focused on potentiating T cell anti-tumor responses.

## Concluding Remarks

It is now clear that cells of the innate immunity, in particular NK cells, play a relevant defensive role in the control of tumor growth and metastases. As shown by many experimental evidences, both *in vitro* and *in vivo*, such anti-tumor effect is related both to direct cytolytic activity and to the production of cytokines that activate other effector cells and promote useful TH1 adaptive responses. Therefore, therapeutic approaches that trigger and/or reconstitute NK cell function and proliferation are crucial in tumor immunotherapy. In addition, NK cells engineered with CAR, targeting tumor antigens, are highly promising. Indeed CAR-NK cells could complement or even replace CAR-T cells in view of their particularly potent cytolytic activity and their peculiar homing capability (70–72). Importantly, in case of loss of the targeted tumor antigen, CAR-NK cells could still exert their anti-tumor activity, particularly in the absence of KIR/HLA ligand matching. In addition, CAR-NK cells, genetically modified to over-express either molecules mediating tumor killing or cytokines able to sustain NK cell proliferation/function (e.g., IL-15), may represent a further valuable tool for adoptive cell therapy of cancer (73, 74). Notably, since NK cells do not cause GvHD, they may be obtained from unrelated donors, thus overcoming major limitations of autologous T cell therapy (time needed for preparation and high costs) and providing a rapid access to an “off-the-shelf” life-saving therapy. Indeed given the possibility to better plan treatments with standardized approaches and appropriate cell numbers, donor-derived allogeneic CAR-NK cells may represent the next generation of cell-based therapies of cancer. Table 2 summarizes recent or ongoing clinical trials based on the use of adoptively infused NK cells.

**Table 2:** Selected recent/ongoing trials of NK-based adoptive therapy of cancer.

**Identifier**	**Trial**	**Status**	**Phase**	**NK cells/drug**	**Tumor**
NCT00900809	QUILT-3.018: Neukoplast™ (NK-92) for the treatment of refractory or relapsed acute myeloid leukemia	Recruitment completed	I	NK-92	Acute myeloid leukemia
NCT03027128	QUILT-3.028: study of haNK™ for infusion in subjects with metastatic or locally advanced solid tumors	Recruitment completed	I	NK-92	Solid tumor
NCT03383978	Intracranial injection of NK-92/5.28.z cells in patients with recurrent HER2-positive glioblastoma (CAR2BRAIN)	Recruiting	I	NK-92/5.28.z	Glioblastoma
NCT02573896	Immunotherapy of relapsed refractory neuroblastoma with expanded NK cells	Recruiting	I	CAR-NK-Ch14.18 lenalidomide	Neuroblastoma
NCT02280525	Cord blood Natural Killer (NK) cells in leukemia/lymphoma	Active, not recruiting	I	NK Cells, lenalidomide, rituximab (anti-CD20), fludarabine, cyclophosphamide, cytarabine	Leukemia
NCT02481934	Clinical trial of expanded and activated autologous NK cells to treat multiple myeloma (NK-VS-MM)	Completed	I	NK Cells, lenalidomide, bortezomib	Multiple myeloma
NCT03415100	Pilot study of NKG2D-ligand targeted CAR-NK cells in patients with metastatic solid tumors	Recruiting	I	CAR-NK cells targeting NKG2D ligands	Solid tumors
NCT01974479	Pilot study of redirected haploidentical natural killer cell infusions for B-lineage acute lymphoblastic leukemia	Suspended	I	Anti-CD19 redirected NK cells	B-cell acute lymphoblastic leukemia
NCT03579927	CAR.CD19-CD28-zeta-2A-iCasp9-IL15-transduced cord blood NK cells, high-dose chemotherapy, and stem cell transplant in treating participants with B-cell lymphoma	Not yet recruiting	I/II	Cord blood-NK Cells Autologous HSCT carmustine Cytarabine, etoposide, filgrastim, melphalan, rituximab (anti-CD20)	CD19 positive, mantle cell lymphoma, recurrent diffuse large B-cell lymphoma, recurrent follicular lymphoma, refractory B-cell non-Hodgkin lymphoma, refractory diffuse large B-Cell lymphoma, refractory follicular lymphoma
NCT03056339	Umbilical and Cord Blood (CB) derived CAR-engineered NK cells for B lymphoid malignancies	Recruiting	I/II	iC9/CAR.19/IL15 transduced CB-NK Cells, fludarabine, cyclophosphamide, mesna, AP1903	B-lymphoid malignancies, acute lymphocytic leukemia, chronic lymphocytic leukemia, non-Hodgkin lymphoma
NCT02839954	CAR-pNK cell immunotherapy in MUC1 positive relapsed or refractory solid tumor	Unknown	I/II	Anti-MUC1 CAR-NK cells	Hepatocellular carcinoma, NSCLc, pancreatic carcinoma, triple-negative invasive breast carcinoma, malignant glioma of brain, colorectal carcinoma, gastric carcinoma
NCT02892695	PCAR-119 bridge immunotherapy prior to stem cell transplant in treating patients with CD19 positive leukemia and lymphoma	Unknown	I/II	Anti-CD19 CAR-NK cells	Acute/chronic lymphocytic leukemia, follicular lymphoma, mantle cell lymphoma, B-cell prolymphocytic leukemia, diffuse large cell lymphoma
NCT02742727	CAR-pNK cell immunotherapy in CD7 positive leukemia and lymphoma	Unknown	I/II	Anti-CD7 CAR-NK cells	Acute myeloid leukemia, precursor T-cell lymphoblastic leukemia-lymphoma, T-cell prolymphocytic leukemia, T-cell large granular lymphocytic leukemia, peripheral T-cell lymphoma, angioimmunoblastic T-cell lymphoma, extranodal NK/T-cell lymphoma, nasal type enteropathy-type intestinal T-cell lymphoma

## Author Contributions

All authors wrote and revised the review.

### Conflict of Interest

The authors declare that the research was conducted in the absence of any commercial or financial relationships that could be construed as a potential conflict of interest. The handling editor declared a shared affiliation, though no other collaboration, with the authors PV, NT, and LM.

## References

[1] Moretta A Bottino C Vitale M Pende D Biassoni R Mingari MC. Receptors for HLA class-I molecules in human natural killer cells. Ann Rev Immunol. (1996) 14:619–48. 10.1146/annurev.immunol.14.1.619 8717527

[2] Moretta A Bottino C Vitale M Pende D Cantoni C Mingari MC. Activating receptors and coreceptors involved in human natural killer cell-mediated cytolysis. Ann Rev Immunol. (2001) 19:197–223. 10.1146/annurev.immunol.19.1.197 11244035

[3] Raulet DH Vance RE. Self-tolerance of natural killer cells. Nat Rev Immunol. (2006) 6:520–31. 10.1038/nri1863 16799471

[4] Vitale M Cantoni C Pietra G Mingari MC Moretta L. Effect of tumor cells and tumor microenvironment on NK-cell function. Eur J Immunol. (2014) 44:1582–92. 10.1002/eji.201344272 24777896

[5] Vacca P Munari E Tumino N Moretta F Pietra G Vitale M Human natural killer cells and other innate lymphoid cells in cancer: friends or foes? Immunol Lett. (2018) 201:14–9. 10.1016/j.imlet.2018.11.004 30439479

[6] Vitale M Cantoni C Della Chiesa M Ferlazzo G Carlomagno S Pende D. A historical overview: the discovery of how NK cells can kill enemies, recruit defense troops, and more. Front Immunol. (2019) 10:1415. 10.3389/fimmu.2019.01415 31316503 PMC6611392

[7] Mariotti FR Quatrini L Munari E Vacca P Moretta L. Innate lymphoid cells: expression of PD-1 and other checkpoints in normal and pathological conditions. Front Immunol. (2019) 10:910. 10.3389/fimmu.2019.00910 31105707 PMC6498986

[8] Croxatto D Martini S Chiossone L Scordamaglia F Simonassi CF Moretta L. IL15 induces a potent antitumor activity in NK cells isolated from malignant pleural effusions and overcomes the inhibitory effect of pleural fluid. Oncoimmunology. (2017) 6:e1293210. 10.1080/2162402X.2017.1293210 28507797 PMC5414879

[9] Rosenberg SA Lotze MT Muul LM Chang AE Avis FP Leitman S A progress report on the treatment of 157 patients with advanced cancer using lymphokine-activated killer cells and interleukin-2 or high-dose interleukin-2 alone. N Engl J Med. (1987) 316:889–97. 10.1056/NEJM198704093161501 3493432

[10] Lotze MT Matory YL Ettinghausen SE Rayner AA Sharrow SO Seipp CA. In vivo administration of purified human interleukin 2. II. Half life, immunologic effects, and expansion of peripheral lymphoid cells *in vivo* with recombinant IL 2. J Immunol. (1985) 135:2865–75. 2993418

[11] Levin AM Bates DL Ring AM Krieg C Lin JT Su L. Exploiting a natural conformational switch to engineer an interleukin-2 'superkine'. Nature. (2012) 484:529–33. 10.1038/nature10975 22446627 PMC3338870

[12] Locatelli F Bauquet A Palumbo G Moretta F Bertaina A. Negative depletion of alpha/beta+ T cells and of CD19+ B lymphocytes: a novel frontier to optimize the effect of innate immunity in HLA-mismatched hematopoietic stem cell transplantation. Immunol Lett. (2013) 155:21–3. 10.1016/j.imlet.2013.09.027 24091162

[13] Bentebibel SE Hurwitz ME Bernatchez C Haymaker C Hudgens CW Kluger HM A first-in-human study and biomarker analysis of NKTR-214, a novel IL2Rbetagamma-biased cytokine, in patients with advanced or metastatic solid tumors. Cancer Discov. (2019) 9:711–21. 10.1158/2159-8290.CD-18-1495 30988166

[14] Romee R Cooley S Berrien-Elliott MM Westervelt P Verneris MR Wagner JE. First-in-human phase 1 clinical study of the IL-15 superagonist complex ALT-803 to treat relapse after transplantation. Blood. (2018) 131:2515–27. 10.1182/blood-2017-12-823757 29463563 PMC5992862

[15] Vallera DA Felices M McElmurry R McCullar V Zhou X Schmohl JU. IL15 trispecific killer engagers (TriKE) make natural killer cells specific to CD33+ targets while also inducing persistence, *in vivo* expansion, and enhanced function. Clin Cancer Res. (2016) 22:3440–50. 10.1158/1078-0432.CCR-15-2710 26847056 PMC4947440

[16] Thaker PH Brady WE Lankes HA Odunsi K Bradley WH Moore KN A phase I trial of intraperitoneal GEN-1, an IL-12 plasmid formulated with PEG-PEI-cholesterol lipopolymer, administered with pegylated liposomal doxorubicin in patients with recurrent or persistent epithelial ovarian, fallopian tube or primary peritoneal cancers: an NRG Oncology/Gynecologic Oncology Group study. Gynecol Oncol. (2017) 147:283–90. 10.1016/j.ygyno.2017.08.001 28802766 PMC5704992

[17] Rosenberg SA Lotze MT Muul LM Leitman S Chang AE Ettinghausen SE. Observations on the systemic administration of autologous lymphokine-activated killer cells and recombinant interleukin-2 to patients with metastatic cancer. N Engl J Med. (1985) 313:1485–92. 10.1056/NEJM198512053132327 3903508

[18] Rosenberg SA Mule JJ Spiess PJ Reichert CM Schwarz SL. Regression of established pulmonary metastases and subcutaneous tumor mediated by the systemic administration of high-dose recombinant interleukin 2. J Exp Med. (1985) 161:1169–88. 10.1084/jem.161.5.1169 3886826 PMC2187617

[19] Tumino N Martini S Munari E Scordamaglia F Besi F Mariotti FR. Presence of innate lymphoid cells in pleural effusions of primary and metastatic tumors: functional analysis and expression of PD-1 receptor. Int J Cancer. (2019) 145:1660–8. 10.1002/ijc.32262 30856277 PMC6767381

[20] Venstrom JM Pittari G Gooley TA Chewning JH Spellman S Haagenson M. HLA-C-dependent prevention of leukemia relapse by donor activating KIR2DS1. N Engl J Med. (2012) 367:805–16. 10.1056/NEJMoa1200503 22931314 PMC3767478

[21] Savani BN Mielke S Adams S Uribe M Rezvani K Yong AS Rapid natural killer cell recovery determines outcome after T-cell-depleted HLA-identical stem cell transplantation in patients with myeloid leukemias but not with acute lymphoblastic leukemia. Leukemia. (2007) 21:2145–52. 10.1038/sj.leu.2404892 17673900

[22] Pende D Spaggiari GM Marcenaro S Martini S Rivera P Capobianco A. Analysis of the receptor–ligand interactions in the natural killer-mediated lysis of freshly isolated myeloid or lymphoblastic leukemias: evidence for the involvement of the poliovirus receptor (CD155) and nectin-2 (CD112). Blood. (2005) 105:2066–73. 10.1182/blood-2004-09-3548 15536144

[23] Dewald JR Fuller DO Muller GC Beier JC. A novel method for mapping village-scale outdoor resting microhabitats of the primary African malaria vector, *Anopheles gambiae*. Malar J. (2016) 15:489. 10.1186/s12936-016-1534-9 27659918 PMC5034649

[24] Cichocki F Cooley S Davis Z DeFor TE Schlums H Zhang B. CD56dimCD57+NKG2C+ NK cell expansion is associated with reduced leukemia relapse after reduced intensity HCT. Leukemia. (2016) 30:456–63. 10.1038/leu.2015.260 26416461 PMC4740203

[25] Cichocki F Taras E Chiuppesi F Wagner JE Blazar BR Brunstein C. Adaptive NK cell reconstitution is associated with better clinical outcomes. JCI Insight. (2019) 4:125553. 10.1172/jci.insight.125553 30674718 PMC6413795

[26] Li Pira G Malaspina D Girolami E Biagini S Cicchetti E Conflitti G. Selective depletion of alphabeta T cells and B cells for human leukocyte antigen-haploidentical hematopoietic stem cell transplantation. A three-year follow-up of procedure efficiency. Biol Blood Marrow Transpl. (2016) 22:2056–64. 10.1016/j.bbmt.2016.08.006 27519279

[27] Pietra G Vitale C Pende D Bertaina A Moretta F Falco M. Human natural killer cells: news in the therapy of solid tumors and high-risk leukemias. Cancer Immunol Immunother. (2016) 65:465–76. 10.1007/s00262-015-1744-y 26289090 PMC11028670

[28] Vacca P Martini S Garelli V Passalacqua G Moretta L Mingari MC NK cells from malignant pleural effusions are not anergic but produce cytokines and display strong antitumor activity on short-term IL-2 activation. Eur J Immunol. (2013) 43:550–61. 10.1002/eji.201242783 23192659

[29] Vacca P Martini S Mingari MC Moretta L. NK cells from malignant pleural effusions are potent antitumor effectors: a clue for adoptive immunotherapy? Oncoimmunology. (2013) 2:e23638. 10.4161/onci.23638 23734317 PMC3654587

[30] Terme M Ullrich E Delahaye NF Chaput N Zitvogel L. Natural killer cell-directed therapies: moving from unexpected results to successful strategies. Nat Immunol. (2008) 9:486–94. 10.1038/ni1580 18425105

[31] Terme M Fridman WH Tartour E. NK cells from pleural effusions are potent antitumor effector cells. Eur J Immunol. (2013) 43:331–4. 10.1002/eji.201243264 23322344

[32] Ni J Miller M Stojanovic A Garbi N Cerwenka A. Sustained effector function of IL-12/15/18-preactivated NK cells against established tumors. J Exp Med. (2012) 209:2351–65. 10.1084/jem.20120944 23209317 PMC3526364

[33] Fyfe G Fisher RI Rosenberg SA Sznol M Parkinson DR Louie AC. Results of treatment of 255 patients with metastatic renal cell carcinoma who received high-dose recombinant interleukin-2 therapy. J Clin Oncol. (1995) 13:688–96. 10.1200/JCO.1995.13.3.688 7884429

[34] Atkins MB Lotze MT Dutcher JP Fisher RI Weiss G Margolin K. High-dose recombinant interleukin 2 therapy for patients with metastatic melanoma: analysis of 270 patients treated between 1985 and 1993. J Clin Oncol. (1999) 17:2105–16. 10.1200/JCO.1999.17.7.2105 10561265

[35] Rosenberg SA. IL-2: the first effective immunotherapy for human cancer. J Immunol. (2014) 192:5451–8. 10.4049/jimmunol.1490019 24907378 PMC6293462

[36] Sim GC Liu C Wang E Liu H Creasy C Dai Z. IL2 variant circumvents ICOS+ regulatory T-cell expansion and promotes NK cell activation. Cancer Immunol Res. (2016) 4:983–94. 10.1158/2326-6066.CIR-15-0195 27697858

[37] Carmenate T Pacios A Enamorado M Moreno E Garcia-Martinez K Fuente D. Human IL-2 mutein with higher antitumor efficacy than wild type IL-2. J Immunol. (2013) 190:6230–8. 10.4049/jimmunol.1201895 23677467

[38] Cooley S He F Bachanova V Vercellotti GM DeFor TE Curtsinger JM. First-in-human trial of rhIL-15 and haploidentical natural killer cell therapy for advanced acute myeloid leukemia. Blood Adv. (2019) 3:1970–80. 10.1182/bloodadvances.2018028332 31266741 PMC6616260

[39] Wrangle JM Velcheti V Patel MR Garrett-Mayer E Hill EG Ravenel JG. ALT-803, an IL-15 superagonist, in combination with nivolumab in patients with metastatic non-small cell lung cancer: a non-randomised, open-label, phase 1b trial. Lancet Oncol. (2018) 19:694–704. 10.1016/S1470-2045(18)30148-7 29628312 PMC6089612

[40] Sarhan D Brandt L Felices M Guldevall K Lenvik T Hinderlie P. 161533 TriKE stimulates NK-cell function to overcome myeloid-derived suppressor cells in MDS. Blood Adv. (2018) 2:1459–69. 10.1182/bloodadvances.2017012369 29941459 PMC6020813

[41] Davis ZB Vallera DA Miller JS Felices M. Natural killer cells unleashed: Checkpoint receptor blockade and BiKE/TriKE utilization in NK-mediated anti-tumor immunotherapy. Sem Immunol. (2017) 31:64–75. 10.1016/j.smim.2017.07.011 28882429 PMC5632228

[42] Gragert L Eapen M Williams E Freeman J Spellman S Baitty R. HLA match likelihoods for hematopoietic stem-cell grafts in the U.S. registry. N Engl J Med. (2014) 371:339–48. 10.1056/NEJMsa1311707 25054717 PMC5965695

[43] Rocha V Locatelli F. Searching for alternative hematopoietic stem cell donors for pediatric patients. Bone Marrow Transpl. (2008) 41:207–14. 10.1038/sj.bmt.1705963 18084331

[44] Pende D Marcenaro S Falco M Martini S Bernardo ME Montagna D. Anti-leukemia activity of alloreactive NK cells in KIR ligand-mismatched haploidentical HSCT for pediatric patients: evaluation of the functional role of activating KIR and redefinition of inhibitory KIR specificity. Blood. (2009) 113:3119–29. 10.1182/blood-2008-06-164103 18945967

[45] Ruggeri L Capanni M Urbani E Perruccio K Shlomchik WD Tosti A. Effectiveness of donor natural killer cell alloreactivity in mismatched hematopoietic transplants. Science. (2002) 295:2097–100. 10.1126/science.1068440 11896281

[46] Falco M Pende D Munari E Vacca P Mingari MC Moretta L. Natural killer cells: from surface receptors to the cure of high-risk leukemia (Ceppellini Lecture). HLA. (2019) 93:185–94. 10.1111/tan.13509 30828978 PMC6767140

[47] Muccio L Falco M Bertaina A Locatelli F Frassoni F Sivori S Late development of FcepsilonRgamma(neg) adaptive natural killer cells upon human cytomegalovirus reactivation in umbilical cord blood transplantation recipients. Front Immunol. (2018) 9:1050 10.3389/fimmu.2018.01050 29868012 PMC5968376

[48] Della Chiesa M Falco M Podesta M Locatelli F Moretta L Frassoni F. Phenotypic and functional heterogeneity of human NK cells developing after umbilical cord blood transplantation: a role for human cytomegalovirus? Blood. (2012) 119:399–410. 10.1182/blood-2011-08-372003 22096237

[49] Locatelli F Merli P Pagliara D Li Pira G Falco M Pende D. Outcome of children with acute leukemia given HLA-haploidentical HSCT after alphabeta T-cell and B-cell depletion. Blood. (2017) 130:677–85. 10.1182/blood-2017-04-779769 28588018

[50] Pistoia V Tumino N Vacca P Veneziani I Moretta A Locatelli F. Human gammadelta T-cells: from surface receptors to the therapy of high-risk leukemias. Front Immunol. (2018) 9:984. 10.3389/fimmu.2018.00984 29867961 PMC5949323

[51] Luyckx A Schouppe E Rutgeerts O Lenaerts C Fevery S Devos T. G-CSF stem cell mobilization in human donors induces polymorphonuclear and mononuclear myeloid-derived suppressor cells. Clinical Immunology. (2012) 143:83–7. 10.1016/j.clim.2012.01.011 22341087

[52] Tay J Levesque JP Winkler IG. Cellular players of hematopoietic stem cell mobilization in the bone marrow niche. Int J Hematol. (2017) 105:129–40. 10.1007/s12185-016-2162-4 27943116

[53] Airoldi I Bertaina A Prigione I Zorzoli A Pagliara D Cocco C. gammadelta T-cell reconstitution after HLA-haploidentical hematopoietic transplantation depleted of TCR-alphabeta+/CD19+ lymphocytes. Blood. (2015) 125:2349–58. 10.1182/blood-2014-09-599423 25612623 PMC4440890

[54] Tumino N Besi F Di Pace AL Mariotti FR Merli P Li Pira G. PMN-MDSC are a new target to rescue graft-versus-leukemia activity of NK cells in haplo-HSC transplantation. Leukemia. (2019). 10.1038/s41375-019-0585-7 31586150 PMC7214239

[55] Moretta F Petronelli F Lucarelli B Pitisci A Bertaina A Locatelli F. The generation of human innate lymphoid cells is influenced by the source of hematopoietic stem cells and by the use of G-CSF. Eur J Immunol. (2016) 46:1271–8. 10.1002/eji.201546079 26840535

[56] Moretta L Bottino C Pende D Vitale M Mingari MC Moretta A. Different checkpoints in human NK-cell activation. Trends Immunol. (2004) 25:670–6. 10.1016/j.it.2004.09.008 15530838

[57] Ishida Y Agata Y Shibahara K Honjo T. Induced expression of PD-1, a novel member of the immunoglobulin gene superfamily, upon programmed cell death. EMBO J. (1992) 11:3887–95. 10.1002/j.1460-2075.1992.tb05481.x 1396582 PMC556898

[58] Pesce S Greppi M Tabellini G Rampinelli F Parolini S Olive D. Identification of a subset of human natural killer cells expressing high levels of programmed death 1: a phenotypic and functional characterization. J Allergy Clin Immunol. (2017) 139:335–46.e3. 10.1016/j.jaci.2016.04.025 27372564

[59] Mariotti FR Petrini S Ingegnere T Tumino N Besi F Scordamaglia F. PD-1 in human NK cells: evidence of cytoplasmic mRNA and protein expression. Oncoimmunology. (2019) 8:1557030. 10.1080/2162402X.2018.1557030 30723590 PMC6350684

[60] Garrido F Aptsiauri N Doorduijn EM Garcia Lora AM van Hall T. The urgent need to recover MHC class I in cancers for effective immunotherapy. Curr Opin Immunol. (2016) 39:44–51. 10.1016/j.coi.2015.12.007 26796069 PMC5138279

[61] Sabbatino F Villani V Yearley JH Deshpande V Cai L Konstantinidis IT. PD-L1 and HLA class I antigen expression and clinical course of the disease in intrahepatic cholangiocarcinoma. Clin Cancer Res. (2016) 22:470–8. 10.1158/1078-0432.CCR-15-0715 26373575 PMC5296951

[62] Munari E Rossi G Zamboni G Lunardi G Marconi M Sommaggio M. PD-L1 assays 22C3 and SP263 are not interchangeable in non-small cell lung cancer when considering clinically relevant cutoffs: an interclone evaluation by differently trained pathologists. Am J Surg Pathol. (2018) 42:1384–9. 10.1097/PAS.0000000000001105 29901568

[63] Munari E Zamboni G Lunardi G Marchionni L Marconi M Sommaggio M. PD-L1 expression heterogeneity in non-small cell lung cancer: defining criteria for harmonization between biopsy specimens and whole sections. J Thorac Oncol. (2018) 13:1113–20. 10.1016/j.jtho.2018.04.017 29704674

[64] Munari E Zamboni G Lunardi G Marconi M Brunelli M Martignoni G. PD-L1 expression in non-small cell lung cancer: evaluation of the diagnostic accuracy of a laboratory developed test using clone E1L3N in comparison with 22C3 and SP263 assays. Hum Pathol. (2019) 90:54–59. 10.1016/j.humpath.2019.05.003 31121194

[65] Andre P Denis C Soulas C Bourbon-Caillet C Lopez J Arnoux T. Anti-NKG2A mAb is a checkpoint inhibitor that promotes anti-tumor immunity by unleashing both T and NK cells. Cell. (2018) 175:1731–43.e13. 10.1016/j.cell.2018.10.014 30503213 PMC6292840

[66] Della Chiesa M Vitale M Carlomagno S Ferlazzo G Moretta L Moretta A. The natural killer cell-mediated killing of autologous dendritic cells is confined to a cell subset expressing CD94/NKG2A, but lacking inhibitory killer Ig-like receptors. Eur J Immunol. (2003) 33:1657–66. 10.1002/eji.200323986 12778484

[67] Mingari MC Ponte M Bertone S Schiavetti F Vitale C Bellomo R HLA class I-specific inhibitory receptors in human T lymphocytes: interleukin 15-induced expression of CD94/NKG2A in superantigen- or alloantigen-activated CD8+ T cells. Proc Natl Acad Sci USA. (1998) 95:1172–7. 10.1073/pnas.95.3.1172 9448304 PMC18710

[68] Bertone S Schiavetti F Bellomo R Vitale C Ponte M Moretta L. Transforming growth factor-beta-induced expression of CD94/NKG2A inhibitory receptors in human T lymphocytes. Eur J Immunol. (1999) 29:23–9. 10.1002/(SICI)1521-4141(199901)29:01<23::AID-IMMU23>3.0.CO;2-Y 9933082

[69] Mingari MC Pietra G Moretta L. Immune checkpoint inhibitors: anti-NKG2A antibodies on board. Trends Immunol. (2019) 40:83–5. 10.1016/j.it.2018.12.009 30609967

[70] Ingegnere T Mariotti FR Pelosi A Quintarelli C De Angelis B Tumino N. Human CAR NK cells: a new non-viral method allowing high efficient transfection and strong tumor cell killing. Front Immunol. (2019) 10:957. 10.3389/fimmu.2019.00957 31114587 PMC6503170

[71] Daher M Rezvani K. Next generation natural killer cells for cancer immunotherapy: the promise of genetic engineering. Curr Opin Immunol. (2018) 51:146–53. 10.1016/j.coi.2018.03.013 29605760 PMC6140331

[72] Rezvani K. Adoptive cell therapy using engineered natural killer cells. Bone Marrow Transpl. (2019) 54(Suppl. 2):785–8. 10.1038/s41409-019-0601-6 31431708 PMC7594488

[73] Quintarelli C Orlando D Boffa I Guercio M Polito VA Petretto A. Choice of costimulatory domains and of cytokines determines CAR T-cell activity in neuroblastoma. Oncoimmunology. (2018) 7:e1433518. 10.1080/2162402X.2018.1433518 29872565 PMC5980417

[74] Sivori S Meazza R Quintarelli C Carlomagno S Della Chiesa M Falco M. NK cell-based immunotherapy for hematological malignancies. J Clin Med. (2019) 8:E1702. 10.3390/jcm8101702 31623224 PMC6832127

